# Compositional regulation of poly(3-hydroxybutyrate-*co*-3-hydroxyhexanoate) by replacement of granule-associated protein in *Ralstonia eutropha*

**DOI:** 10.1186/s12934-015-0380-8

**Published:** 2015-11-23

**Authors:** Yui Kawashima, Izumi Orita, Satoshi Nakamura, Toshiaki Fukui

**Affiliations:** Department of Bioengineering, Graduate School of Bioscience and Biotechnology, Tokyo Institute of Technology, 4259 Nagatsuta, Midori-ku, Yokohama, 226-8501 Japan

**Keywords:** Polyhydroxyalkanoates, *Ralstonia eutropha*, PHA synthase, Phasin

## Abstract

**Background:**

Phasin (PhaP), a kind of polyhydroxyalkanoate (PHA) granule-associated proteins, has a role in controlling the properties of PHA granules surface, and is thought to have influence on PHA biosynthesis in PHA-producing bacteria. This study focused on the *phaP1*_*Re*_ locus in *Ralstonia eutropha* as a site of chromosomal modification for production of flexible poly(3-hydroxybutyrate-*co*-3-hydroxyhexanoate) [P(3HB-*co*-3HHx)] from soybean oil.

**Results:**

Considering the high expression level of *phaP1*_*Re*_, *phaJ*_*Ac*_ [encoding (*R*)-specific enoyl-CoA hydratase from *Aeromonas caviae*] was inserted into the downstream of *phaP1*_*Re*_ on chromosome 1 of *R. eutropha* strain NSDG harboring *phaC*_NSDG_ (encoding PHA synthase with broad substrate specificity). The constructed strain efficiently accumulated P(3HB-*co*-3HHx) on soybean oil with higher 3HHx composition when compared to the previous strain having *phaJ*_*Ac*_ within *pha* operon. Insertion of the second *phaC*_NSDG_ along with *phaJ*_*Ac*_ at the *phaP1*_*Re*_ locus led to incorporation of much larger 3HHx fraction into PHA chains, although the molecular weight was markedly reduced. The *R. eutropha* strains were further engineered by replacing *phaP1*_*Re*_ with *phaP*_*Ac*_ (encoding phasin from *A. caviae*) on the chromosome. Interestingly, the phasin replacement increased 3HHx composition in the soybean oil-based PHA with keeping high cellular contents, nevertheless no modification was conducted in the metabolic pathways. Kinetic and Western blot analyses of PHA synthase using cellular insoluble fractions strongly suggested that the phasin replacement not only enhanced activity of PHA synthase from *A. caviae* but also increased affinity especially to longer (*R*)-3HHx-CoA. It was supposed that the increased affinity of PHA synthase to (*R*)-3HHx-CoA was responsible for the higher 3HHx composition in the copolyester.

**Conclusions:**

The downstream of *phaP1*_*Re*_ was a useful site for integration of genes to be overexpressed during PHA accumulation in *R. eutropha*. The results also clarified that polymerization properties of PHA synthase was affected by the kind of phasin co-existed on the surface of PHA granules, leading to altered composition of the resulting P(3HB-*co*-3HHx). The phasin replacement is a novel engineering strategy for regulation of composition of PHA copolyesters.

**Electronic supplementary material:**

The online version of this article (doi:10.1186/s12934-015-0380-8) contains supplementary material, which is available to authorized users.

## Background

Polyhydroxyalkanoates (PHAs) are biopolyesters produced by a number of microorganisms as intracellular carbon and energy storage materials. PHAs have attracted industrial attentions as one of possible solutions for recent environmental problems caused by general petroleum-based plastics, because PHAs are eco-friendly polymeric materials that can be produced from renewable biomass resources, and show biodegradable and biocompatible properties [1, 2].

**Fig. 1:**
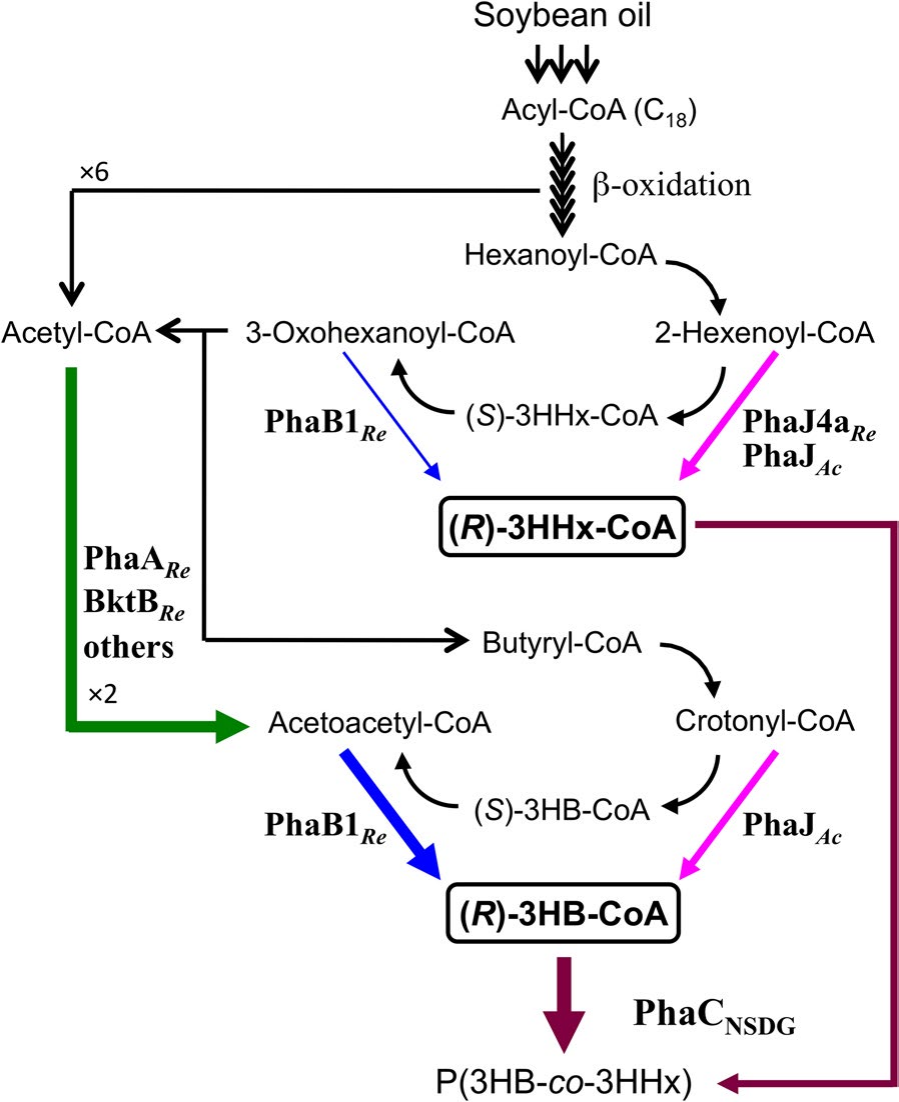
Proposed P(3HB-*co*-3HHx) biosynthesis pathway from soybean oil through β-oxidation by *R. eutropha* NSDG-based recombinant strains. *PhaA*_*Re*_ and *BktB*_*Re*_ β-ketothiolases, *PhaB1*_*Re*_ NADPH-acetoacetyl-CoA reductase, *PhaJ*_*Ac*_ and *PhaJ4a*_*Re*_ (*R*)-specific enoyl-CoA hydratases, *FadB*_*Re*_ bifunctional (*S*)-specific 2-enoyl-CoA hydratase/(*S*)-3-hydroxyacyl-CoA dehydrogenase, *PhaC*_*NSDG*_ N143S/D171G mutant of PHA synthase from *A. caviae*, *Re R. eutropha*, *Ac A. caviae*

The most abundant PHA in nature is poly(3-hydroxybutyrate) [P(3HB)], which is generally synthesized from acetyl-CoA through three consecutive reactions by β-ketothiolase (PhaA), NADPH-dependent acetoacetyl-CoA reductase (PhaB) and PHA synthase (PhaC). *Ralstonia eutropha* (*Cupriavidus necator*) H16 is a well-studied P(3HB) producer [3, 4]. It has been known that the three genes for P(3HB) biosynthesis are clustered as *phaC*-*A*-*B1*_*Re*_ operon, and PhaC_*Re*_ shows polymerization activity toward short-chain-length (*R*)-3-hydroxyacyl (3HA)-CoAs of C_3_–C_5_. However, the application range of P(3HB) is very limited due to the brittle and struggle properties attributed to the high crystallinity and high melting temperature. Poly(3-hydroxybutyrate-*co*-3-hydroxyhexanoate) [P(3HB-*co*-3HHx)] is a PHA copolyester naturally produced by some bacteria such as *Aeromonas caviae* from vegetable oils and fatty acids [5]. This copolyester has been demonstrated to show more soft and flexible properties that are suitable for practical applications when compared to P(3HB) homopolymer [1, 5]. In *A. caviae*, (*R*)-3HA-CoA monomers of C_4_ and C_6_ are provided from 2-enoyl-CoA intermediates in β-oxidation by the function of (*R*)-specific enoyl-CoA hydratase (PhaJ_*Ac*_), and successively polymerized by PhaC_*Ac*_ having unique substrate specificity (C_4_-C_7_) [6, 7]. Based on these facts, efforts have been made to construct recombinant bacteria for efficient production of P(3HB-*co*-3HHx). Several previous studies engineered *R. eutropha* H16 by introduction of heterologous PHA synthase and (*R*)-specific enoyl-CoA hydratase, resulting in biosynthesis of P(3HB-*co*-3HHx) composed of adequately high 3HHx fractions from vegetable oils (Fig. 1) [8–12]. Tsuge et al. have reported that the Asn149Ser/Asp171Gly double mutant of *A. caviae* PHA synthase, named PhaC_NSDG_, could accept more 3HHx unit than wild type PhaC_*Ac*_ in vivo [8]. Insomphan et al. reported that deletion of one of *fadB* homologs in *R. eutropha* harboring *phaC*_NSDG_ increased 3HHx fractions in the soybean oil-based P(3HB-*co*-3HHx) [13]. Recently, an artificial pathway for synthesis of this copolyester from structurally unrelated fructose was developed in *R. eutropha* [14].

In PHA-accumulating bacterial cells, PHA chains are gathered and make granules in cytoplasm, and the PHA granules are covered with various proteins called PHA granule-associated proteins, PGAPs [15, 16]. Phasins (PhaPs) are amphiphilic small size proteins and known to be major PGAPs widely distributed in various PHA producers [17, 18]. The functions of PhaPs are thought to not only control the properties of PHA granules surface, but also have influence on PHA biosynthesis. PhaP1_*Re*_ is the most abundant phasin in *R. eutropha* H16 [17]. The expression level is very high owing to the strong *phaP1* promoter [19, 20], by which transcription is regulated by PHA granule-binding transcriptional factor PhaR_*Re*_ [15, 21]. PhaP1_*Re*_ occupied approximately 5 % of the total proteins in the crude extract of the cells cultivated on fructose [17], and was estimated to cover 27–54 % of surface of the PHA granules [22]. It was demonstrated that PhaP1_*Re*_ participated in control of the size and number of intracellular PHA granules and consequent amount of PHA, as the *phaP1*_*Re*_-deficient strain of *R. eutropha* stored approximately half amount of P(3HB) as many smaller granules in comparison with the wild strain [17, 23]. Interestingly, PhaP1_*Re*_ did not bind to PhaC1_*Re*_ directly in two-hybrid assay [24], but formed a high-molecular weight complex along with PhaC1_*Re*_ and soluble P(3HB) oligomer in *R. eutropha* [25]. The PhaC1_*Re*_-PhaP1_*Re*_-PHB complex showed no lag phase in PhaC activity assay, which suggested that PhaC_*Re*_ had an active form in the complex.

*Aeromonas caviae* also has phasin (PhaP_*Ac*_) of which gene is organized as *phaP*-*C*-*J*_*Ac*_ operon on the chromosome [6]. PhaP_*Ac*_ belongs to a different class of phasin from PhaP1_*Re*_, as they share no significant homology. When *phaPCJ*_*Ac*_ genes were highly expressed in *A. caviae*, a numerous number of small PHA granules were accumulated within the cells [26]. Moreover, a recombinant strain of *A. caviae* overexpressing *phaPC*_*Ac*_ synthesized P(3HB-*co*-3HHx) with much higher 3HHx composition (46–52 mol %) than the strain overexpressing *phaC*_*Ac*_ alone (16–21 mol %) [26]. Although the detail for this phenomenon has not been elucidated, the result suggested some effects of PhaP_*Ac*_ on catalytic properties of PhaC_*Ac*_. Recent in vitro analyses demonstrated that DNA/PHA-binding protein PhaM_*Re*_ activated polymerization activity of PhaC1_*Re*_, and formed a high-molecular-weight complex with PhaC1_*Re*_ [27]. Another enzyme assay indicated that PhaP1_*Re*_ and PhaP_*Ac*_ inhibited PhaC1_*Re*_ activity, whereas they enhanced the activity of PhaC_*Ac*_ up to 2.4 to 3-fold [28].

In this study, we focused on the *phaP1*_*Re*_ locus as a site for chromosomal modification in *R. eutropha* for production of P(3HB-*co*-3HHx). The effects of insertion of PHA biosynthesis genes at the downstream of *phaP1*_*Re*_, as well as those of replacement of *phaP* on biosynthesis of PHA copolyester from soybean oil were investigated.

## Results

### Effects of insertion of *phaJ*_*Ac*_/*phaC*_NSDG_ at downstream of *phaP1*_*Re*_ on PHA biosynthesis

**Fig. 2:**
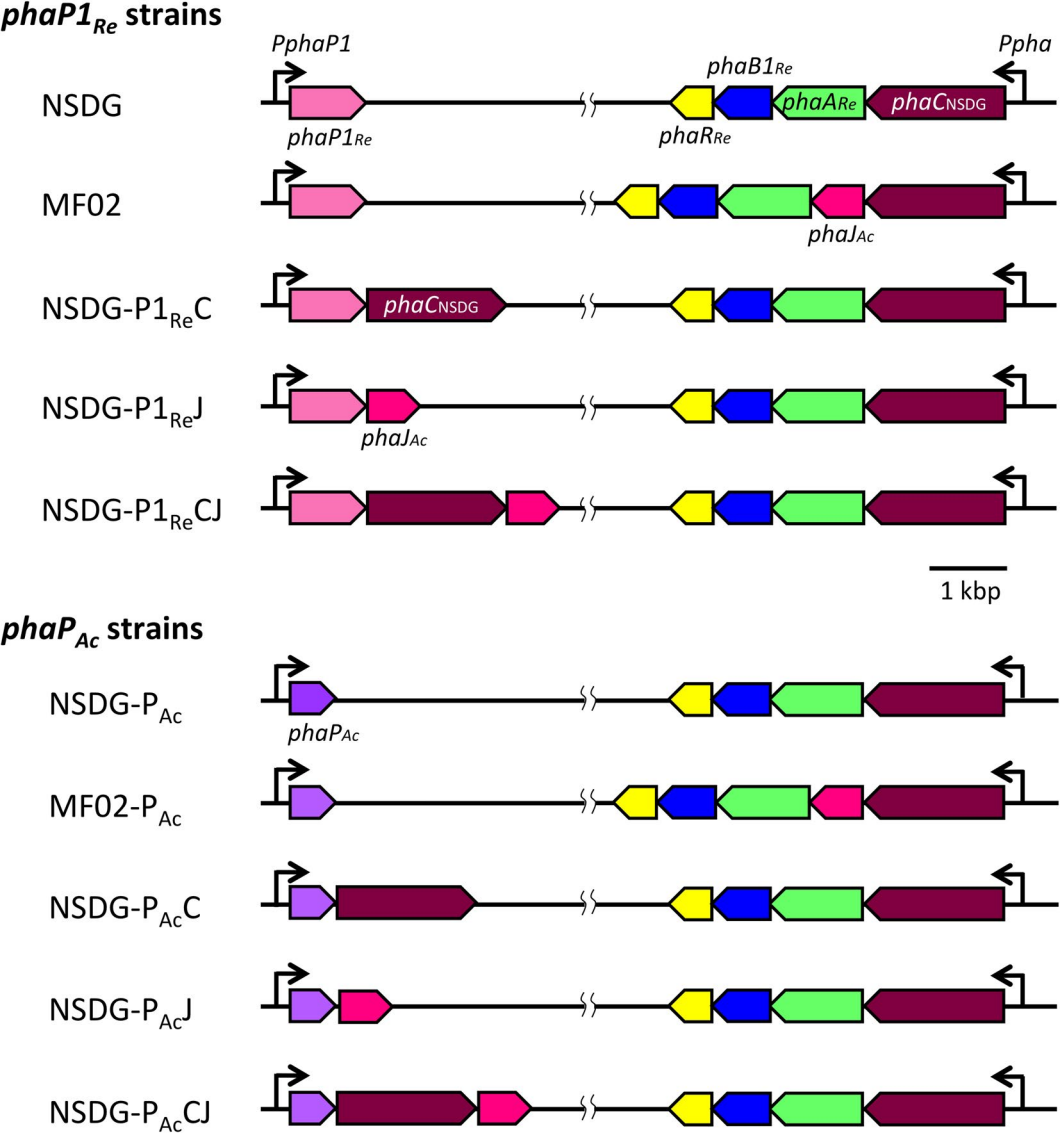
Schematic diagram of genotypes of *R. eutropha* NSDG-based recombinant strains. *phaA*_*Re*_, β-ketothiolase gene; *phaB1*_*Re*_, NADPH-acetoacetyl-CoA reductase gene; *phaR*_*Re*_, a gene encoding PHA-binding transcriptional repressor; *phaJ*_*Ac*_, (*R*)-specific enoyl-CoA hydratase gene; *phaC*_NSDG_, a gene encoding N143S/D171G mutant of PHA synthase from *A. caviae*; *P*_*phaP1*_ and *P*_*pha*_, promoter regions of *phaP1*_*Re*_ and *phaCAB1*_*Re*_ in *R. eutropha*, respectively. *Re R. eutropha*, *Ac A. caviae*

Previous studies have demonstrated that introduction of PhaJ capable of accepting the C_6_-substrate was effective to increase 3HHx composition of P(3HB-*co*-3HHx) produced by *R. eutropha* from vegetable oils [10–12]. While, the expression of *phaP1*_*Re*_, regulated by PhaR_*Re*_ [15, 21], is highly induced at the PHA accumulation phase and the induced transcription level is one of the highest in *R. eutropha* H16 [19, 20]. We here examined insertion of *phaJ*_*Ac*_ and/or the second copy of *phaC*_NSDG_ into the downstream of *phaP1*_*Re*_ on chromosome 1 of *R. eutropha* strain NSDG possessing *phaC*_NSDG_ instead of native *phaC*_*Ac*_ in *pha* operon. The gene organization in the constructed strains NSDG-P1_Re_C, NSDG-P1_Re_J, and NSDG-P1_Re_CJ are illustrated in Fig. 2.

These strains were cultivated in a nitrogen-limited synthetic medium containing 1 % (v/v) soybean oil as a sole carbon source, and the results of PHA biosynthesis are shown in Table 1. The insertion of the additional copy of *phaC*_NSDG_ at the downstream of *phaP1*_*Re*_ gave almost no influence on production and composition of PHA. NSDG-P1_Re_J, constructed by insertion of *phaJ*_*Ac*_, produced similar amount of P(3HB-*co*-3HHx) with fourfold higher 3HHx composition compared to the parent strain NSDG. This increase of 3HHx fraction was slightly larger than that observed by the strain MF02 harboring *phaJ*_*Ac*_ within *pha* operon. Enzyme assay indicated that enoyl-CoA hydratase activities toward crotonyl-CoA (C_4_) and 2-hexenoyl-CoA (C_6_) in NSDG-P1_Re_J (C_4_: 27.2 U/mg, C_6_: 12.0 U/mg) were approximately 3.0- and 2.3-fold higher than those in MF02 (C_4_: 9.1 U/mg, C_6_: 5.2 U/mg).

**Table 1:** PHA biosynthesis by *R. eutropha* recombinant strains from soybean oil

Strain	Dry cell mass (g/L)	PHA content (wt%)	PHA (g/L)	3HHx composition (mol %)
(*phaP1*_*Re*_ strains)				
NSDG	6.9 ± 0.02	86 ± 4.3	5.9	1.6 ± 0.03
NSDG-P1_Re_C	6.7 ± 0.21	91 ± 0.3	6.0	1.8 ± 0.10
NSDG-P1_Re_J	6.7 ± 0.05	89 ± 1.5	6.0	6.5 ± 0.09
NSDG-P1_Re_CJ	5.7 ± 0.18	84 ± 1.3	4.8	10.5 ± 0.26
MF02	6.8 ± 0.02	88 ± 0.51	6.0	4.6 ± 0.07
(*phaP*_*Ac*_ strains)				
NSDG-P_Ac_	7.1 ± 0.18	89 ± 1.1	6.3	2.0 ± 0.05
NSDG-P_Ac_C	6.4 ± 0.11	88 ± 1.5	5.6	2.1 ± 0.51
NSDG-P_Ac_J	7.0 ± 0.40	87 ± 1.1	6.1	8.8 ± 0.24
NSDG-P_Ac_CJ	6.3 ± 0.07	79 ± 3.3	5.0	17.2 ± 0.18
MF02-P_Ac_	7.2 ± 0.30	90 ± 1.2	6.5	6.4 ± 0.10

The cells were cultivated in an MB medium containing 1 % (v/v) soybean oil at 30 °C for 72 h (n = 3)

When *phaC*_NSDG_-*phaJ*_*Ac*_ genes were inserted at the site, the resulting strain NSDG-P1_Re_CJ accumulated P(3HB-*co*-3HHx) with 3HHx fraction of 10.5 mol % (Table 1). This was 6.6-fold larger than that in the copolymer synthesized by the strain NSDG, although the PHA production was decreased from 5.9 to 4.8 g/l.

### Effects of replacement of *phaP1*_*Re*_ by *phaP*_*Ac*_ on PHA biosynthesis

*phaP1*_*Re*_ on chromosome 1 was replaced by *phaP*_*Ac*_ from *A. caviae* in the recombinant strains of *R. eutropha* expressing *phaC*_NSDG_ (Fig. 2), in order to investigate the change of PHA biosynthesis profiles by phasin derived from the same source as PHA synthase. Interestingly, this genetic modification tended to increase 3HHx composition without serious reduction of PHA production from soybean oil in all the strains examined, as shown in Table 1. The compositional change was noticeable in the strains expressing *phaJ*_*Ac*_ and the additional *phaC*_NSDG_ at the downstream of *phaP*, as the 3HHx fraction in P(3HB-*co*-3HHx) significantly increased to 17.2 mol % in NSDG-P_Ac_CJ compared to 10.5 mol % in NSDG-P1_Re_CJ. The 3HHx compositions of the copolymers synthesized by NSDG-P_Ac_J and MF02-P_Ac_ (6.4–8.8 mol %) were also higher than those in the corresponding strains having native PhaP1_*Re*_ (4.6–6.5 mol %). The effect of the phasin replacement was not remarkable in the strains not expressing *phaJ*_*Ac*_, such as NSDG and NSDG-P1_*Re*_C.

### Molecular weights of PHAs accumulated in *R. eutropha* recombinant strains

The number-average-molecular weights (*M*_n_) of P(3HB-*co*-3HHx) synthesized by the *phaP*-replaced strains NSDG-P_Ac_ and NSDG-P_Ac_J were 1.8- and 1.4-fold higher than those by the corresponding parent strains NSDG and NSDG-P1_Re_J, respectively (Table 2). The results suggested that PhaP_*Ac*_ allowed PhaC_NSDG_ co-existed on the granule surface to synthesize longer PHA chain than PhaP1_*Re*_. The strains NSDG-P1_Re_CJ and NSDG-P_Ac_CJ accumulated PHA with drastically decreased *M*_n_ to approximately one-ninth of those obtained by the strains not having the second copy of *pha*C_NSDG_. The values of polydispersity index of the resulting PHAs were not changed by the replacement of phasin.

**Table 2:** Molecular weights of PHA synthesized by *R. eutropha* recombinant strains from soybean oil

Strain	PhaP	*M*_n_ × 10^5^	*M*_w_ × 10^5^	PDI
NSDG	PhaP1_*Re*_	7.5 ± 3.1	24.4 ± 5.23	3.56 ± 0.80
NSDG-P_Ac_	PhaP_*Ac*_	13.5 ± 2.3	42.3 ± 5.93	3.16 ± 0.26
NSDG-P1_Re_J	PhaP1_*Re*_	10.0 ± 2.1	33.8 ± 6.05	3.52 ± 0.90
NSDG-P_Ac_J	PhaP_*Ac*_	13.8 ± 1.2	48.1 ± 2.16	3.52 ± 0.25
NSDGP1_Re_CJ	PhaP1_*Re*_	1.2 ± 0.17	2.8 ± 0.25	2.28 ± 0.21
NSDG-P_Ac_CJ	PhaP_*Ac*_	1.5 ± 0.36	3.6 ± 1.39	2.35 ± 0.32

PHA was extracted and purified from the cells cultivated in an MB medium containing 1 % (v/v) soybean oil at 30 °C for 72 h (n = 3). *M*_*n*_ number-average molecular weight, *M*_*w*_ weight-average molecular weight, *PDI* polydispersity index (*M*_w_/*M*_n_)

### Kinetic parameters of PHA synthase on the surface of PHA granules

**Fig. 3:**
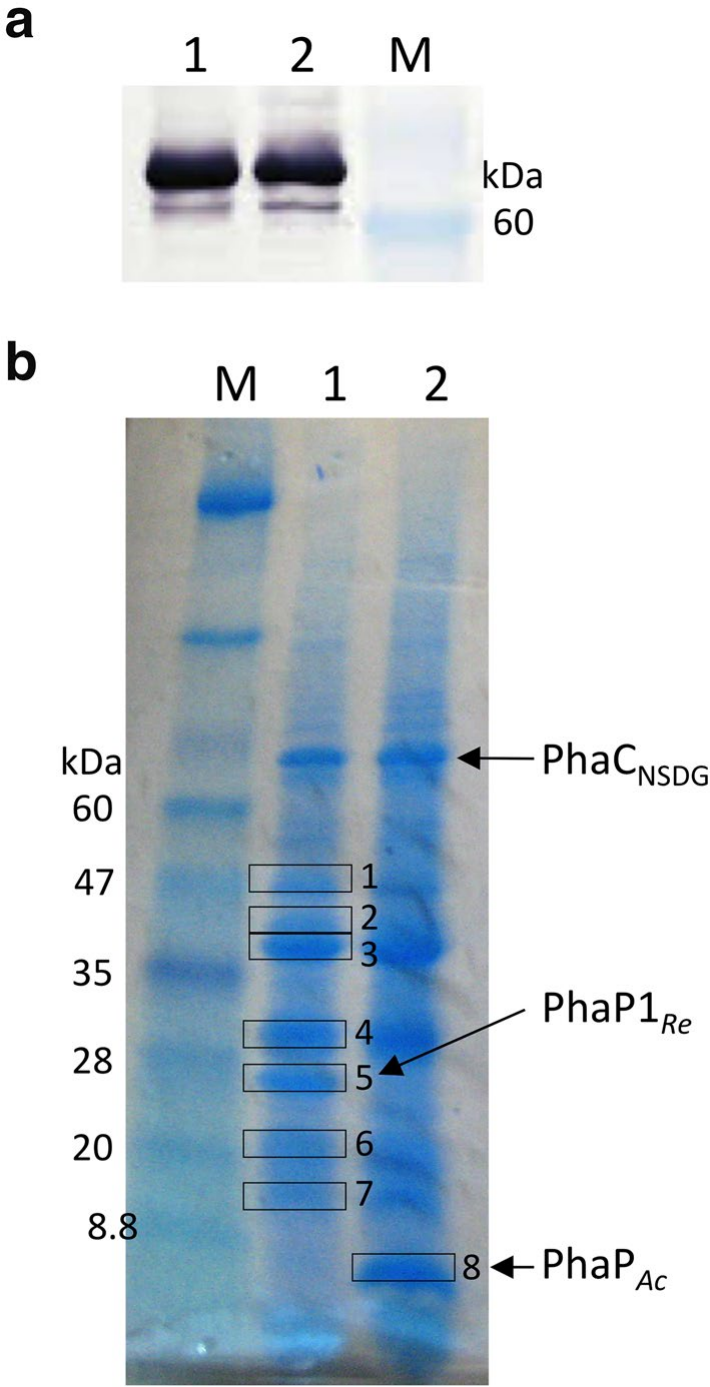
**a** Western blot analysis of insoluble fractions (12.5 µg) of *R. eutropha* strains using anti-PhaC_*Ac*_ antiserum. **b** SDS-PAGE analysis of PHA granule fractions (700 µg of dry granules). *Lanes*: *M* protein marker; 1, *R. eutropha* strain NSDG-P1_Re_CJ; 2, *R. eutropha* strain NSDG-P_Ac_CJ. The *boxed* bands (no. 1–8) in **b** were subjected to protein identification by LC–MS/MS

PHA synthase and phasin were bound on the surface of PHA granules contained in a cellular insoluble fraction. The kinetic analysis of PhaC_NSDG_ on PHA granules was done using the insoluble fraction of *R. eutropha* to investigate the effects of the phasin replacement on the catalytic properties of PhaC_NSDG_. We initially attempted the analysis for the strains NSDG and NSDG-P_Ac_ grown on soybean oil, however, PHA synthase activity of the insoluble fractions was too low to determine reliable parameters. Therefore, the strains NSDG-P1_Re_CJ and NSDG-P_Ac_CJ, both harboring the second copy of *phaC*_NSDG_, were used for the sources of the insoluble fraction. When the polymerization activities of PhaC_NSDG_ were determined toward (*R*)-3HB-CoA (C_4_) and (*R*)-3HHx-CoA (C_6_), no lag-phase of the absorbance change was observed. The reactions followed Michaelis–Menten kinetics, and the determined kinetic parameters are shown in Table 3. PHA synthases in both the strains showed lower activity to (*R*)-3HHx-CoA than to (*R*)-3HB-CoA. Interestingly, the replacement of PhaP1_*Re*_ by PhaP_*Ac*_ led to 2.6 to 2.8-fold increase in the V_max_ values, and significantly reduced the K_m_ values to one-fourth to one-tenth. The C_6_/C_4_ ratios of V_max_ were almost the same as each other for the two synthase samples, while the C_6_/C_4_ ratio of K_m_ was markedly decreased from 1.9 with PhaP1_*Re*_ (NSDG-P1_Re_CJ) to 0.71 with PhaP_*Ac*_ (NSDG-P_Ac_CJ). These results indicated that the increase of affinity of PhaC_NSDG_ by the phasin replacement was more significant to the longer C_6_-substrate. Consequently, the C_6_/C_4_ ratio of V_max_/K_m_ of the synthase in the *phaP*_*Ac*_-expressing strain was 3.0-fold larger than that in the strain expressing native *phaP1*_*Re*_, which was consistent with the increase of the 3HHx fraction in the resulting PHA. The amounts of the PhaC_NSDG_ protein in the insoluble fractions of NSDG-P1_Re_CJ and NSDG-P_Ac_CJ, determined by Western blot analysis, were similar level as shown in Fig. 3a, indicating that the differences in the kinetic parameters were not due to changes in the amount of PHA synthase on the granule surface. We also observed that (*R*)-specific enoyl-CoA hydratase activity to the C_4_- and C_6_-substrates were not different between the soluble fractions of the two strains (C_4_: 14.3–15.3 U/mg and C_6_: 1.1–1.3 U/mg, respectively). The similar amounts of PhaC_NSDG_ and activities of PhaJ_*Ac*_ in the two strains indicated little influence of the replacement of *phaP* on transcription driven by the upstream promoter region.

**Table 3:** Kinetic parameters of PhaC_NSDG_ in insoluble fractions of *R. eturopha* recombinant strains toward (*R*)-3HB-CoA (C_4_) and (*R*)-3HHx-CoA (C_6_)

Strain (genotype)	Substrate chain length	K_m_		V_max_		V_max_/K_m_	
		(µM)	C_6_/C_4_ ratio	(µmol/min/mg-insoluble^a^)	C_6_/C_4_ ratio		C_6_/C_4_ ratio
NSDG-P1_Re_CJ	C_4_	320	1.9	0.21	0.20	6.4 × 10^−4^	0.10
(*phaP1*_*Re*_-*phaC*_NSDG_-*phaJ*_*Ac*_)	C_6_	614		0.041		6.6 × 10^−5^	
NSDG-P_Ac_CJ	C_4_	86	0.71	0.52	0.21	6.1 × 10^−3^	0.30
(*phaP*_*Ac*_-*phaC*_NSDG_-*phaJ*_*Ac*_)	C_6_	61		0.11		1.8 × 10^−3^	

The insoluble fractions were prepared from the cells cultivated in an MB medium containing 1 % (v/v) soybean oil at 30 °C for 48 h

^a^Dry weight of the insoluble fraction

### Confirmation of phasin replacement and identification of proteins in PHA granule fractions

The proteins in PHA granule fractions of the strains NSDG-P1_Re_CJ and NSDG-P_Ac_CJ, prepared by density-gradient ultracentrifugation, were separated by SDS-PAGE (Fig. 3b), and the major eight protein bands were subjected to LC–MS/MS analysis for identification. The details of the results are shown in Additional file 1: Table S1. The 24-kDa protein (no. 5) in NSDG-P1_Re_CJ was determined to be PhaP1_*Re*_. In the strain NSDG-P_Ac_CJ, this band was disappeared and a smaller protein (no. 8) in large abundance, not present in NSDG-P1_Re_CJ, was identified to be PhaC_*Ac*_. This result clearly indicated actual replacement of major phasin on PHA granules to PhaP_*Ac*_ in NSDG-P_Ac_CJ. The protein band no. 3 was outer membrane protein (porin), and the bands of no. 1, 2, 4, 6, and 7 contained multiple kinds of proteins including various membrane proteins, ribosomal proteins, and hypothetical proteins. Several proteins involved in important cellular functions were also detected, such as elongation factor Tu and cell division protein FtsA in the no. 1 band and RNA polymerase subunit α in the no. 2 band. These proteins were considered to be the result of contamination of other cell fractions, but possibility of the presence on the surface as PGAPS could not be excluded, as observed and discussed previously [16, 31]. Although *R. eutropha* possesses many phasins [3, 24, 29, 30], only PhaP5_*Re*_ could be detected in the band no. 7 besides PhaP1_*Re*_ in NSDG-P1_Re_CJ.

### Electron microscopy

**Fig. 4:**
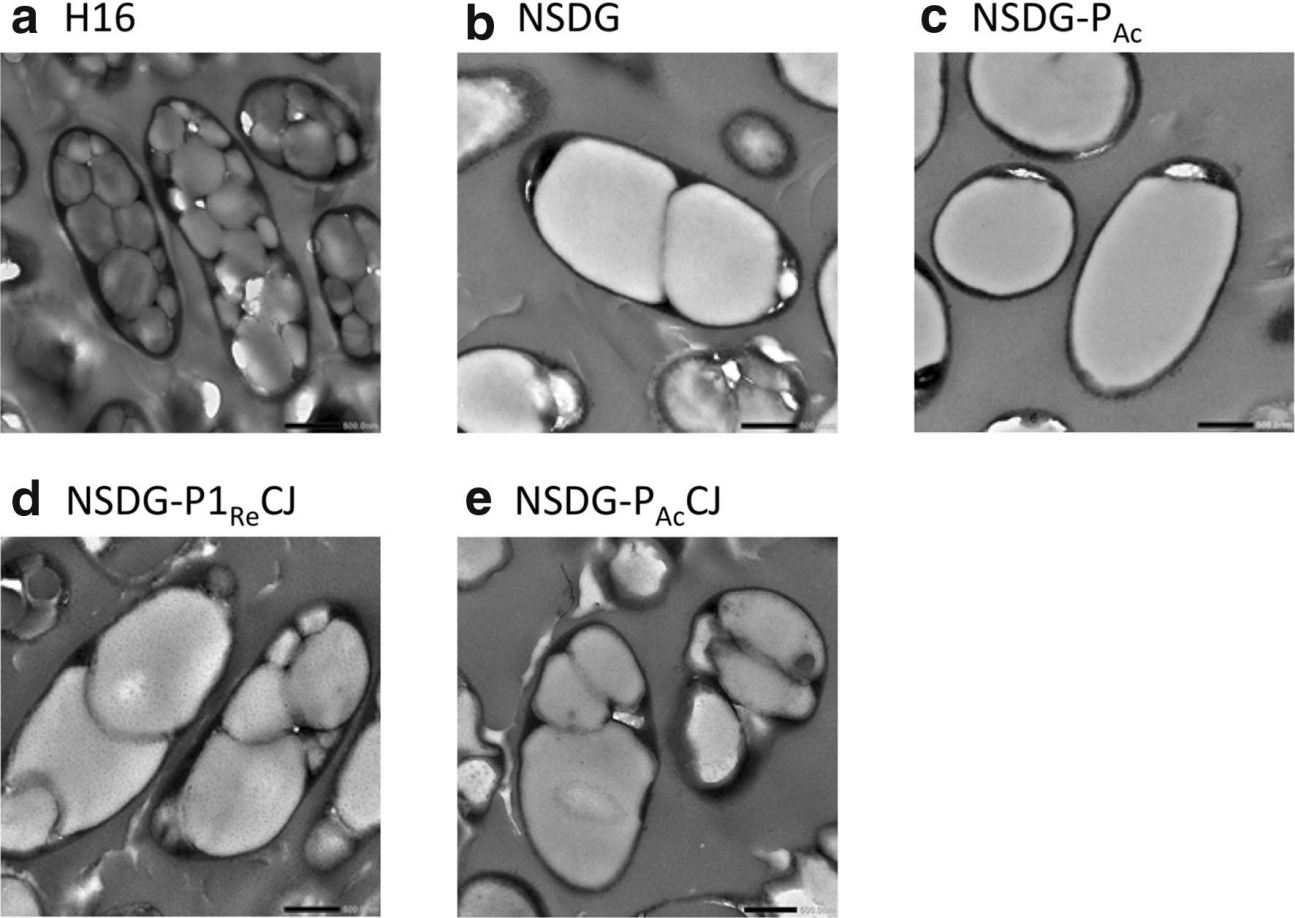
Transmission electron micrographs of ultrathin sections of *R. eutropha* strains. Magnification: ×10,000; *scale bar* 0.5 μm

The accumulation of PHA granules within *R. eutropha* H16, NSDG, NSDG-P_Ac_, NSDG-P1_Re_CJ, and NSDG-P_Ac_CJ grown on soybean oil were observed by transmission electron microscopy (Fig. 4). The wild strain H16 accumulated 10–16 of small PHA granules per one cell, whereas NSDG and NSDG-P_Ac_ formed one to three of much larger PHA granule(s). In the cells of NSDG-P1_Re_CJ and NSDG-P_Ac_CJ, granules with various sizes were existed together. The size and number of PHA granules were greatly affected by the kind of PHA synthase, but not by phasin on the granule surface. The reason for these observations has been unclear yet, but the formation of less larger granules in the strain NSDG was similar to phenotype of the *phaP1*_*Re*_-disruptant [17]. The hydrophilicity of cytosolic side of the PhaC_NSDG_ protein on PHA granules may be lower than that of PhaC_*Re*_, leading to formation of large granules with reduced surface area.

## Discussion

The gene of the major phasin in *R. eutropha* H16, *phaP1*_*Re*_, is one of the most highly expressed genes at the PHA accumulation phase [19, 20]. Barnard et al. have achieved T7 promoter-driven high level production of recombinant proteins by using the *phaP1*_*Re*_ locus as the integration site for T7 RNA polymerase [32]. We here applied the downstream of *phaP1*_*Re*_ as an insertion site for PHA biosynthesis-related gene(s) in *R. eutropha*, because the induction profile and expression level of *phaP1*_*Re*_ higher than *phaCAB1*_*Re*_ were expected to be advantage for PHA biosynthesis. When *phaC*_NSDG_ was solely inserted at the site in the strain NSDG, no change in P(3HB-*co*-3HHx) composition was observed, probably due to poor provision of (*R*)-3HHx-CoA through the intact channeling pathway from β-oxidation to PHA biosynthesis. Indeed, enhancement of the channeling pathway by expression of *phaJ*_*Ac*_ significantly increased 3HHx composition without impairment of PHA production, as observed previously [10, 12]. The results of the cultivation suggested that the expression level of the gene located in the *phaP1*_*Re*_ locus was higher than within *pha* operon, given the higher 3HHx composition in the resulting P(3HB-*co*-3HHx) and higher enoyl-CoA hydratase activity in NSDG-P1_Re_J (*phaP1*_*Re*_-*phaJ*_*Ac*_) than those in the strain MF02 (*phaC*_NSDG_-*phaJ*_*Ac*_-*phaAB1*_*Re*_). The insertion of the second copy of *phaC*_NSDG_ at the downstream of *phaP1*_*Re*_ led to drastic decrease of the molecular weight of PHA by less than one-tenth. Previous studies [33–35] reported a negative correlation of intracellular PhaC activity or *phaC* expression level with P(3HB) molecular weight, and one of possible reasons for this phenomenon may be due to large number of catalytic molecules (PhaC_NSDG_) relative to substrate molecules [(*R*)-3HA-CoAs]. The present study was consistent with the previous observations, and the similar effects of plasmid-borne multi copies of *phaC* on P(3HB-*co*-3HHx) biosynthesis has been also seen in recombinant *R. eutropha* grown on plant oils [11].

**Fig. 5:**
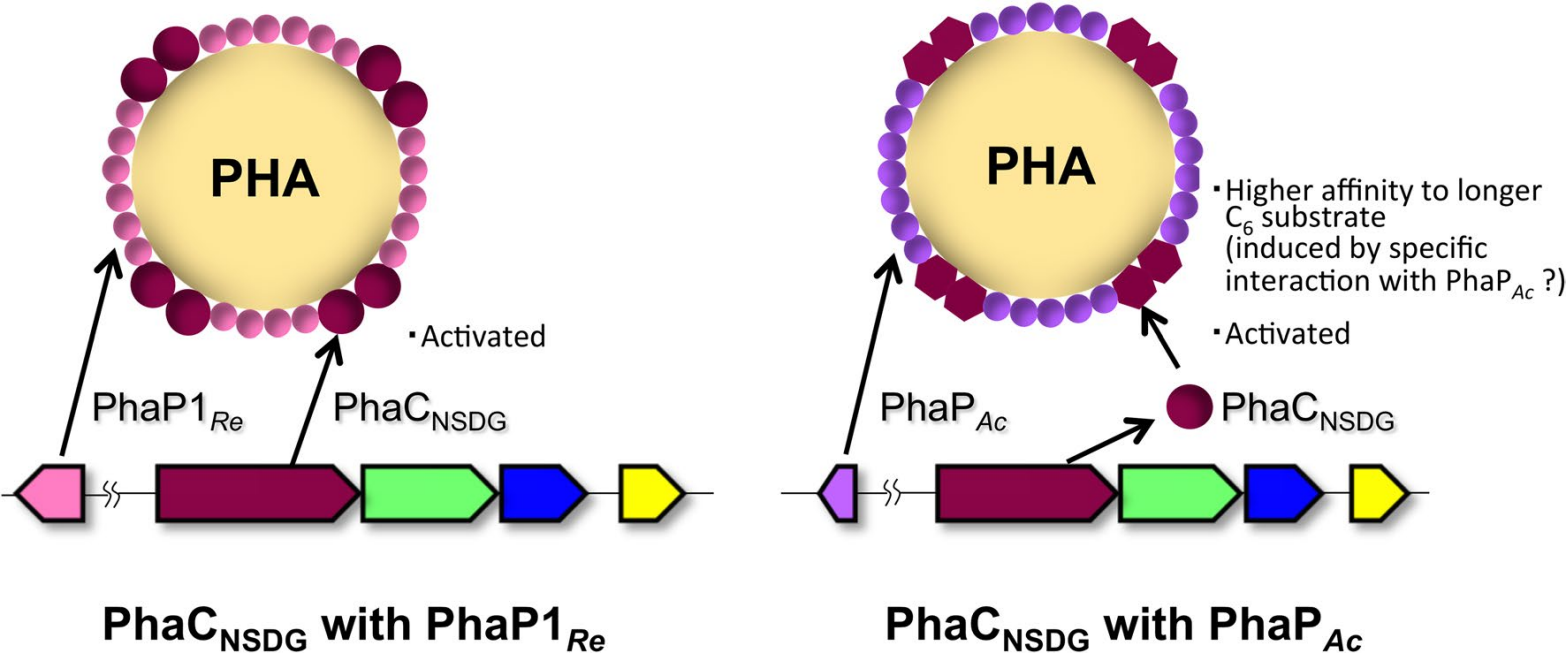
Estimated situations of the surface of PHA granule in *R. eutropha* strains expressing *phaC*_NSDG_ together with *phaP1*_*Re*_ (*left*) or *phaP*_*Ac*_ (*right*)

This study also demonstrated a novel engineering strategy for compositional regulation of P(3HB-*co*-3HHx); the phasin replacement. We previously observed that overexpression of *phaC*_*Ac*_ together with *phaP*_*Ac*_ in *A. caviae* significantly increased the 3HHx fraction in PHA produced from fatty acids when compared to overexpression of *phaC*_*Ac*_ alone [26]. One of possible explanation for this phenomenon was some changes of catalytic properties of PhaC_*Ac*_ in the presence of PhaP_*Ac*_ derived from the same source. However, such compositional change had not been observed when *phaPC*_*Ac*_ were introduced in *R. eutropha* PHB^−^4 [6]. We assumed that, even if PhaP_*Ac*_ affected catalytic properties of PhaC_*Ac*_, the effects might be disturbed by the presence of large amount of native phasins covering the surface of PHA granules in *R. eutropha*. The *phaP1*_*Re*_ encoding major phasin was thus replaced by *phaP*_*Ac*_ by homologous recombination, and the results demonstrated that the phasin replacement tended to increase 3HHx fraction in P(3HB-*co*-3HHx) with keeping high PHA contents on soybean oil. Further kinetic analysis of PhaC_NSDG_ using native granules clarified interesting changes of the catalytic properties depending on the origin of phasin. The replacement of PhaP1_*Re*_ by PhaP_*Ac*_ significantly increased the catalytic efficiency of PhaC_NSDG_ that had been already active form on the PHA granule surface. It has been reported that both PhaP_*Ac*_ and PhaP1_*Re*_ enhanced the polymerization activity of PhaC_*Ac*_ toward (*R*)-3HB-CoA in in vitro assay using the free proteins not associated with PHA granules, where the activation by PhaP_*Ac*_ was slightly stronger than that by PhaP1_*Re*_ [28]. It should be noted that the enhancement of polymerization activity by the replacement of PhaP1_*Re*_ with PhaP_*Ac*_ on the native granules (2.5 to 2.7-fold) was much larger than that observed in the case using the free proteins (1.3-fold). In addition, the phasin replacement more specifically increased the affinity of PhaC_NSDG_ toward the longer (*R*)-3HHx-CoA, as seen in the increase of C_6_/C_4_ ratio of the K_m_ values (Table 3). It was feasible that the higher relative affinity of the polymerizing enzyme to the C_6_-substrate was responsible for the larger 3HHx fraction in PHA synthesized by the strain NSDG-P_Ac_CJ. Ushimaru et al. proposed that phasin helps to withdraw a PHA chain from PhaC_*Ac*_ exhibiting lower turnover rate than PhaC1_*Re*_, and thus prevent the chain from aggregation that potentially blocks the release site in the active dimer [28]. This idea can well explain the activation of PhaC_*Ac*_ by phasins, but not enough to understand the great increase of the substrate affinity of PhaC_NSDG_, particularly to the C_6_-substrate, by the phasin replacement. Although the detailed mechanism has been remained to be cleared, it was supposed that phasin could interact with active PHA synthase on the granule surface. In the case of specific combination of PHA synthase and phasin such as PhaC_NSDG_ and PhaP_*Ac*_, the interaction might induce some structural change potentially affecting the affinity and specificity toward the substrates into PHA synthase (Fig. 5). The molecular weights of the accumulated PHA also tended to be increased by the phasin replacement. The higher activity of PhaC_NSDG_ in the presence of PhaP_*Ac*_ on the granule surface would lead formation of longer polymer chains than the enzyme with PhaP1_*Re*_.

The expression of *phaJ* was very effective for increasing 3HHx composition in P(3HB-*co*-3HHx) synthesized from fatty acids and plant oils by recombinant *R. eutropha* strains [10–12]. However, we previously observed saturation of the provision of 3HHx unit by this strategy [12]. Another strategy for increasing 3HHx composition was suppression of 3HB unit formation by disruption of *phaA* encoding thiolase, although this accompanied reduction of PHA production [10]. The advantage of the present phasin replacement strategy was further increase of 3HHx composition even in the *phaJ*-expressing strains without impairment of PHA biosynthesis. The *R. eutropha* strains applying this novel strategy can efficienty produce P(3HB-*co*-3HHx) composed of 9–17 mol % 3HHx fractions, which was suitable for practical applications with adequate flexibility [5].

## Conclusion

This study demonstrated that the downstream of *phaP1*_*Re*_ on chromosome 1 of *R. eutropha* was a useful site for integration of PHA biosynthesis genes to achieve the overexpression during the PHA accumulation phase. In addition, we found that replacement of the major phasin PhaP1_*Re*_ in recombinant *R. eutropha* by PhaP_*Ac*_ from *A. caviae* resulted in increase of 3HHx composition of P(3HB-*co*-3HHx) without serious decrease of the productivity on soybean oil.

A general engineering strategy for alteration of PHA copolymer composition has been modification of metabolic flux of monomer-supplying pathways. The phasin replacement is a novel strategy not directly modifying metabolic pathways, thus there is no need to consider unexpected negative effects on global metabolisms. It was also shown that catalytic properties of PHA synthase were affected by phasin co-existed on the surface of PHA granules, and significance of the effects appeared to be depended on the kind of phasin. These results were interesting in understanding the role of phasin in PHA biosynthesis. In terms of the application, the use of adequate pair of PHA synthase and phasin would allow us to obtain PHA copolymers with desired composition with high productivity.

## Methods

### Bacterial strains and plasmids

The bacterial strains and plasmids used in this study are listed in Additional file 1: Table S2. *R. eutropha* strains were cultivated at 30 °C in a nutrient-rich (NR) medium containing 10 g of meat extract, 10 g of polypeptone, 2 g of yeast extract in 1 L of tap water. *E. coli* strains were cultivated at 37 °C in a Lysogeny broth (LB) medium. Kanamycin (100 mg/L) or ampicillin (50 mg/L) was added to the medium when necessary.

### Construction of plasmids and strains

DNA manipulations were carried out according to standard procedures, and the sequences of oligonucleotide primers used for PCR are shown in Additional file 1: Table S3. KOD-Plus-ver.2 or KOD-Plus-Neo DNA polymerase and Ligation High ver.2 (Toyobo, Otsu, Japan) were used for PCR amplification and ligation, respectively.

The upstream and downstream regions flanked to *phaP1*_*Re*_ were individually amplified by PCR from *R. eutropha* H16 genomic DNA as a template with primer sets of phaP_Re_out-Fw/phaP_Re_-Inv1 and phaP_Re_out-Rv/phaP_Re_-Inv2, respectively. The latter fragment was 5′-phosphorylated, ligated with the former, and the upstream–downstream fragment was subjected to the second PCR using phaP_Re_out-Fw/phaP_Re_out-Rv primers. The amplified upstream–downstream fusion was inserted into pK18mobsacB at the SmaI site, leading to construction of pK18mobsacB-ΔP1.

The vectors for replacement of *phaP1*_*Re*_ by *phaP*_*Ac*_, *phaP*_*Ac*_-*phaC*_NSDG_, *phaP*_*Ac*_-*phaC*_NSDG_-*phaJ*_*Ac*_, or *phaPJ*_*Ac*_ were constructed as follows. The coding region of *phaC*_NSDG_ was amplified from pTA2-NSDG [10] by PCR using a primer set of pEE32R-C_Ac_up-inv/pEE32R-C_Ac_down-inv. A linear fragment not containing *phaC*_*Ac*_ in pEE32 harboring *phaPCJ*_*Ac*_ [6] was prepared by inverse PCR with phaP_Re_-Inv1/phaP_Re_-Inv2 primers, and then ligated with the *phaC*_NSDG_ fragment to obtain pEE32-NSDG harboring *phaP*_*Ac*_-*C*_NSDG_-*J*_*Ac*_. The fragments of *phaP*_*Ac*_, *phaP*_*Ac*_-*C*_NSDG_, and *phaP*_*Ac*_-*C*_NSDG_-*J*_*Ac*_ were amplified with pEE32-NSDG as a template and primer sets of phaP_Ac_-Fw/phaP_Ac_-Rv, phaP_Ac_-Fw/phaC_NSDG_-Rv, and phaP_Ac_-Fw/phaJ_Ac_-Rv, respectively. The amplified fragments were 5′-phosphorylated and then individually inserted between upstream and downstream regions of *phaP1*_*Re*_ by ligation with an inverse PCR product obtained with pK18mobsacB-ΔP1 as a template and phaP_Re_-Inv1/phaP_Re_-Inv2 primers. The resulting plasmids were designated pK18mobsacB-P_Ac_, pK18mobsacB-P_Ac_C, and pK18mobsacB-P_Ac_CJ, respectively. pK18mobsacB-P_Ac_J was constructed by inverse PCR for elimination of the *phaC*_NSDG_ region from pK18mobsacB-P_Ac_CJ with a primer set phaP_Ac_down-Inv/phaJ_Ac_-Fw, followed by self-ligation after 5′-phosphorylation.

pK18mobsacB-P1_Re_C, pK18mobsacB-P1_Re_J and pK18mobsacB-P1_Re_CJ, used for insertion of the genes from *A. caviae* at downstream of *phaP1*_*Re*_, were constructed from pK18mobsacB-P_Ac_C, pK18mobsacB-P_Ac_J and pK18mobsacB-P_Ac_CJ, respectively. Linear fragments not having the *phaP*_*Ac*_ region were prepared from these plasmids by inverse PCR using phaP_Re_-Inv1/phaP_Ac_-down-Inv primers. The coding region of *phaP1*_*Re*_ was amplified with *R. eutropha* H16 genome DNA and a primer set of phaP_Re_-Fw/phaP_Re_-Rv, and the *phaP1*_*Re*_ fragment was ligated with the corresponding inverse PCR product with the same direction as the *phaP1* promoter.

All the pK18mobsacB-based plasmids were transferred from *E. coli* S17-1 by conjugation to *R. eutropha* strains NSDG or MF02, and the recombinant strains formed by pop-in-pop-out recombination were selected, according to the procedures reported previously [10].

### PHA production by recombinant *R. eutropha* strains

PHA production by *R. eutropha* strains was carried out on a reciprocal shaker (115 strokes/min) at 30 °C in a 500 ml flask with a 100 ml of a nitrogen-limited mineral salts (MB) medium and 0.1 ml of trace-element solution [36]. Soybean oil was directly added to the medium at 1.0 % (v/v) as a sole carbon source. After the cultivation for 72 h, the cells were harvested, washed with cold 70 % ethanol, washed again with deionized water, and then lyophilized. The cellular PHA content and monomer composition were determined by gas chromatography (GC) after methanolysis of the dried cells in the preference of 15 % sulfuric acid [35]. Extraction and purification of the accumulated PHA from the dried cells, and determination of molecular weight by gel permutation chromatography (GPC) were performed as described previously [37].

### Enzyme assay

Crotonyl-CoA was synthesized by condensation of crotonic anhydride (Tokyo Chemical Industry, Tokyo, Japan) with lithium salt of CoA-SH [38]. Chemical synthesis of (*R*)-3HB-CoA and *trans*-2-hexenoyl-CoA by a mixed-anhydride method, and enzymatic conversion of *trans*-2-hexenoyl-CoA to (*R*)-3HHx-CoA were performed as described previously [39], except for the use of recombinant PhaJ4a_*Re*_ [12] as (*R*)-specific enoyl-CoA hydratase.

*Ralstonia eutropha* strains were cultivated for 48 h in an MB medium at 30 °C with 1.0 % (v/v) soybean oil as a carbon source. The grown cells were harvested, washed by Tris–HCl buffer (pH 7.5), and then disrupted by high pressure homogenization as described previously [36]. The disrupted cells were centrifuged (18,800*g*, 10 min, 4 °C) to separate the soluble fraction and insoluble fraction containing native PHA granules. PHA synthase activity of the insoluble fraction was assayed with the reaction mixture composed 0.05–0.5 mM (*R*)-3HB-CoA or (*R*)-3HHx-CoA, 1 mM 5,5′-dithiobis (2-nitrobenzoic acid) [DTNB] in 50 mM phosphate buffer (pH 7.2), and 10 µl of the insoluble fraction. The increase in absorbance at 412 nm corresponding to release of free CoA-SH was measured spectrophotometrically at 30 °C (ε_412_ = 14.5 × 10^3^). Enoyl-CoA hydratase activity, including both (*S*)- and (*R*)-specific activities, in the soluble fraction was determined in the reaction mixture composed of 250 µM crotonyl-CoA or *trans*-2-hexenoyl-CoA in 50 mM Tris–HCl (pH 8.0) and 5 µl of the soluble fraction. The hydration of the enoyl-CoA substrates were monitored as decrease in absorbance at 263 nm at 30 °C (ε_263_ = 6.7 × 10^3^ M^−1^ cm^−1^). (*R*)-specific enoyl-CoA hydratase activity in the soluble fraction was determined by the hydration of the enoyl-CoA substrates coupled with polymerization of the resulting (*R*)-3-hydroxyalyl-CoAs of C_4_ and C_6_ by using the insoluble fraction of *R. eutropha* NSDG-P_Ac_C as a source of PHA synthase. The reaction mixture was composed of 0.20 mM *trans*-2-enoyl-CoA, 1 mM DTNB in 50 mM phosphate buffer (pH 8.0), 5 µl of the insoluble fraction of the strain NSDG-P_Ac_C, and the increase of absorbance at 412 nm was monitored at 30 °C.

The aliquot of the insoluble fraction was lyophilized to determine the dry weight, and used to calculate specific activities. The proteins contained in 12.5 µg of the insoluble fraction were separated by sodium dodecylsulfate polyacrylamide gel electrophoresis (SDS-PAGE) with 15 % acrylamide gel, according to the standard procedure. Western blot analysis for PhaC_NSDG_ was performed using a specific antiserum against PhaC_*Ac*_ [26]. A goat anti-rabbit IgG (Fc fragment specific)-alkaline phosphatase conjugate (Calbiochem, CA, USA) was used as the secondary antibody, and 1-Step NBT/BCIP plus suppressor (Thermo Fisher Scientific, MA, USA) was used to detect the signals according to the manufacture’s instruction. Protein concentration of the soluble fraction was determined by the method of Bradford with bovine serum albumin as the standard.

### Transmission electron microscope

The cells of the *R. eutropha* strains cultivated in an MB medium with 1 % (v/v) soybean oil at 30 °C for 48 h were harvested, washed, and resuspended in 0.1 M phosphate buffer (pH 7.2) containing 0.1 M sucrose. The cells were fixed with 2.5 % (w/v) glutaraldehyde in 0.1 M phosphate buffer (pH 7.2) containing 0.1 M sucrose, and subsequently with 1 % (w/v) osmium tetroxide in the buffer. The fixed cells were dehydrated in a graded ethanol and embedded with epoxy resin Quetol 651 (Nissin EM, Tokyo, Japan). The ultrathin sections (80 nm) of the embedded cells were prepared with a Leica UC7 ultramicrotome. The sections on a copper grid were stained with EM stainer (Nissin EM) followed by Reynolds’s lead citrate at room temperature. The specimens were observed on a JEOL 1400Plus transmission electron microscope with an accelerating voltage of 100 kV.

### Isolation of native PHA granule fractions and identification of proteins

The insoluble fractions of *R. eutropha* strains were loaded on the top of a discontinuous glycerol gradient of 2 mL of 88 % glycerol and 8 mL of 44 % glycerol in 100 mM Tris–HCl (pH 7.5). After the ultracentrifugation (210,000*g*, 40 min, 4 °C), the PHA granules at the interphase of 44–88 % glycerol were collected, following by washing with 100 mM Tris–HCl (pH 7.5). The granules resuspended in 1 mL of the buffer were subsequently loaded on a discontinuous sucrose gradient prepared with 1 mL of 1.8 M sucrose, 2.9 mL of 1.6, 1.4, and 1.2 M sucrose, and 1 mL of 1.0 M sucrose in the buffer, and then subjected to ultracentrifugation (210,000*g*, 2 h, 4 °C). The band of the PHA granules were observed at the interphase of 1.2–1.4 M sucrose for P(3HB-*co*-3HHx) composed of 10.5 mol % 3HHx (produced by the strain NSDG-P1_Re_CJ), and that of 1.0–1.2 M sucrose for P(3HB-*co*-3HHx) composed of 17.2 mol % 3HHx (produced by the strain NSDG-P_Ac_CJ). The bands of PHA granules were recovered, washed, and resuspended in 100 mM Tris–HCl (pH 7.5).

The PHA granule suspension containing approximately 700 μg of dried inclusion was separated by SDS-PAGE with 4–15 % gradient gel (Bio-Rad, Hercules, CA, USA). The tryptic peptides were prepared from the SDS-PAGE gel, and subjected to mass spectrometric analysis using an LC–MS system equipped with Acquity UPLC apparatus (Waters, Milford, MA, USA) and Synapt High Definition Mass Spectrometer (Waters). LC was performed with a reversed-phase C_18_ column (75 µm × 150 mm, 1.7 µm particle size) with the mobile phase of acetonitrile gradient in 1 % formic acid at the flow rate of 0.5 μl/min. The mass spectrometry was done in the positive ion mode. The MS/MS spectra were analyzed by Protein Lynx Global Server (Waters).

## Additional file

10.1186/s12934-015-0380-8 **Table S1**. Identification of proteins in PHA granule fractions from *R. eutropha* recombinant strains grown on soybean oil. **Table S2**. Bacterial strains and plasmids used in this study. **Table S3**. Sequences of primers used in this study.
