# Supplementary material for: Compositional regulation of poly(3-hydroxybutyrate-co-3-hydroxyhexanoate) by replacement of granule-associated protein in Ralstonia eutropha
Source: Microb Cell Fact. 2015 Nov 23;14:187. doi: 10.1186/s12934-015-0380-8 (PMC4657207; doi:10.1186/s12934-015-0380-8)
Supplement: Supplementary file 1 — 10.1186/s12934-015-0380-8 Table S1. Identification of proteins in PHA granule fractions from R. eutropha recombinant strains grown on soybean oil. Table S2. Bacterial strains and plasmids used in this study. Table S3. Sequences of primers used in this study. [file 12934_2015_380_MOESM1_ESM.pdf]

Supplementary Information

Compositional Regulation of Poly(3-hydroxybutyrate-*co*-3-hydroxyhexanoate) by Replacement of Granule-associated Protein in *Ralstonia eutropha*

Yui Kawashima, Izumi Orita, Satoshi Nakamura, Toshiaki Fukui*

Department of Bioengineering, Graduate School of Bioscience and Biotechnology, Tokyo Institute of Technology, 4259 Nagatsuta, Midori-ku, Yokohama 226-8501, Japan

*Corresponding author
Mailing address: tfukui@bio.titech.ac.jp

Supplementary Table S1. Identification of proteins in PHA granule fractions from *R. eutropha* recombinant strains grown on soybean oil.

Sample no.	Locus_tag (gene)	Annotation	size (kDa)
1	H16_A0342 (*ctaC*)	Cytochrome c oxidase subunit 2	45.8
	H16_A1205	Uncharacterized lipoprotein	44.7
	H16_A2361	Outer membrane protein assembly factor BamB	41.6
	H16_A3491 (*tufA*)	Elongation factor Tu (EF-Tu)	43.3
	H16_A3269 (*ftsA*)	Cell division protein	44.5
2	H16_A0083	Outer membrane protein (Porin)	38.9
	H16_A3284	Outer membrane protein (Porin)	40.1
	H16_A3688	TRAP-type transporter, periplasmic component	41.8
	H16_A0935	ABC-type transporter, periplasmic component	41.2
	H16_A3233	Uncharacterized protein	38.4
	H16_A3458 (*rpoA*)	DNA-directed RNA polymerase subunit alpha (RNA polymerase subunit alpha, Transcriptase subunit alpha)	35.7
	H16_A0544 (*recA*)	Recombinase A	37.7
3	H16_A3402	Outer membrane protein (Porin)	41.3
4	H16_A2221 (*htpX*)	Zn-dependent protease with chaperone function	28.2
	H16_A3500 (*rplA*)	50S ribosomal protein L1	24
	H16_A3484 (*rplC*)	50S ribosomal protein L3	22.8
	H16_A0475	ABC-type transporter, ATPase component: PAAT family	27.4
	H16_A1433	Outer membrane protein assembly factor BamD	31.5
	H16_A3459 (*rpsD*)	30S ribosomal protein S4	23.4
	H16_A2977	Predicted periplasmic or secreted protein	24.4
	H16_A2629 (*sdhB*)	Succinate dehydrogenase (Fe-S protein subunit)	27.4
	H16_A1439 (*phaB1*)	Acetoacetyl-CoA reductase	26.5
5	H16_A1381 (*phaP1*)	Phasin (PHA-granule associated protein)	20.0
6	H16_A0495	Uncharacterized protein	19.9
	H16_B1248	Bacterial DNA-binding protein, histone-like	15.9
	H16_A3641 (*atpF*)	ATP synthase subunit b (ATP synthase F(0) sector subunit b) (ATPase subunit I) (F-ATPase subunit b)	17.3
	H16_A1040	Uncharacterized protein containing LysM domain	17.7
	H16_B2304	Hypothetical lipoprotein	21.6
7	H16_A0495	Uncharacterized protein	19.9
	H16_B2202	Uncharacterized protein	20.6
	H16_A3641 (*atpF*)	ATP synthase subunit b (ATP synthase F(0) sector subunit b) (ATPase subunit I) (F-ATPase subunit b)	17.3
	H16_B1934 (*phaP5*)	Uncharacterized protein	15.7
8	$PhaP_{Ac}$ from *A. caviae*	Phasin (PHA-granule associated protein)	12.6

The PHA granule fractions were prepared from the cells grown in MB medium containing 1% (v/v) soybean at 30°C for 72 h.

Supplementary Table S2. Bacterial strains and plasmids used in this study

Strain or plasmid	Relevant marker(s)	Reference
Strains		
Escherichia coli		
S17-1	*thi pro hsdR recA* chromosomal RP4; Tra^+; Tmp^r St^r/Spc^r	(1)
Ralstonia eutropha		
NSDG	H16 derivative; Δ$phaC_{Re}$::$phaC_{NSDG}$	(2)
NSDG-$P1_{Re}J$	NSDG derivative; $phaP1_{Re}$-$phaJ_{Ac}$	This study
NSDG-$P1_{Re}C$	NSDG derivative; $phaP1_{Re}$-$phaC_{NSDG}$	This study
NSDG-$P1_{Re}CJ$	NSDG derivative; $phaP1_{Re}$-$phaC_{NSDG}$-$phaJ_{Ac}$	This study
NSDG-P_{Ac}	NSDG derivative; Δ$phaP1_{Re}$::$phaP_{Ac}$	This study
NSDG-$P_{Ac}J$	NSDG derivative; Δ$phaP1_{Re}$::$phaP_{Ac}$-$phaJ_{Ac}$	This study
NSDG-$P_{Ac}C$	NSDG derivative; Δ$phaP1_{Re}$::$phaP_{Ac}$-$phaC_{NSDG}$	This study
NSDG-$P_{Ac}CJ$	NSDG derivative; Δ$phaP1_{Re}$::$phaP_{Ac}$-$phaC_{NSDG}$-$phaJ_{Ac}$	This study
MF02	NSDG derivative; $phaJ_{Ac}$	(2)
MF02-P_{Ac}	MF02 derivative; Δ$phaP1_{Re}$::$phaP_{Ac}$	This study
Plasmids		
pEE32	pUC18 derivative; $phaPCJ_{Ac}$	(3)
pTA2NSDG	pTA2 derivative; $phaC_{NSDG}$	(2)
pEE32-NSDG	pEE32 derivative; $phaP_{Ac}$-$phaC_{NSDG}$-$phaJ_{Ac}$	This study
pK18mobsacB	pMB1 ori, *mob*, Kan^r, *sacB*	(4)
pK18mobsacBΔP1	pK18mobsacB derivative; $phaP1_{Re}$ *del*	This study
pK18mobsacB-P_{Ac}	pK18mobsacB derivative; Δ$phaP1_{Re}$::$phaP_{Ac}$	This study
pK18mobsacB-$P1_{Re}J$	pK18mobsacB derivative; $phaP1_{Re}$-$phaJ_{Ac}$	This study
pK18mobsacB-$P1_{Re}C$	pK18mobsacB derivative; $phaP1_{Re}$-$phaC_{NSDG}$	This study
pK18mobsacB-$P1_{Re}CJ$	pK18mobsacB derivative; $phaP1_{Re}$-$phaC_{NSDG}$-$phaJ_{Ac}$	This study
pK18mobsacB-$P_{Ac}J$	pK18mobsacB derivative; Δ$phaP1_{Re}$::$phaP_{Ac}$-$phaJ_{Ac}$	This study
pK18mobsacB-$P_{Ac}C$	pK18mobsacB derivative; Δ$phaP1_{Re}$::$phaP_{Ac}$-$phaC_{NSDG}$	This study
pK18mobsacB-$P_{Ac}CJ$	pK18mobsacB derivative; Δ$phaP1_{Re}$::$phaP_{Ac}$-$phaC_{NSDG}$-$phaJ_{Ac}$	This study

del: homologous regions for deletion the target gene by homologous recombination

Supplementary Table S3. Sequences of primers used in this study

Primer	Sequence (5'-3')
pEE32R-C$_{Ac}$down-inv	GCGCACAATCCCTGGAAGTAGGCCAGA
pEE32R-C$_{Ac}$up-inv	GTGCTCTCCTTCACCCACACCCGA
phaC$_{NSDG}$-Fw	ATGAGCCAACCATCTTATGGCCC
phaC$_{NSDG}$-Rv	GCGGCGTCCTCCTCTGTTGGGCAGGCAA
phaP$_{Re}$out-Fw	CGGGATCCCTGGTGCACATCCAGGTCGACCACG
phaP$_{Re}$out-Rv	CGGGATCCGACGCGTTCTATGTTGCCTTCAC
phaP$_{Re}$-Inv1	TGCTGGTCTCCAGTGGTGAACTTC
phaP$_{Re}$-Inv2	TAACTGCCTGCGTTGAAGATGGAC
phaP$_{Ac}$-Fw	ATGAATATGGACGTGATCAAGAGCTTT
phaP$_{Ac}$-Rv	TCAGGCCTTGCCCGTGCTTTTCTTGATG
phaJ$_{Ac}$-Rv	TCGACGCGGCCGCTTCGAAACTAGTTTAAGGCAGCTTGACCACGGCTTCCC
phaP$_{Ac}$down-Inv	TAACCCCTGGCTGCCCGTTCGGGCAGCCACATCTCCCCAT
phaP1$_{Re}$-Fw	ATGATCCTCACCCCGGAACAA
phaP1$_{Re}$-Rv	TCAGGCAGCCGTCGTCTTCTTTGCCGT

Under lines: restriction sites

References

1. Simon R, Priefer U, Pühler A: A broad host range mobilization system for *in vivo* genetic engineering. Transposon mutagenesis in gram negative bacteria. Bio/Technology 1983, **1**:784-791.

2. Mifune J, Nakamura S, Fukui T: Engineering of *pha* operon on *Cupriavidus necator* chromosome for efficient biosynthesis of poly(3-hydroxybutyrate-*co*-3-hydroxyhexanoate) from vegetable oil. *Polym Degrad Stab* 2010, **95**:1305–1312.

3. Fukui T, Doi Y: Cloning and analysis of the poly (3-hydroxybutyrate-*co*-3-hydroxyhexanoate) biosynthesis genes of *Aeromonas caviae*. *J Bacteriol* 1997, **179**:4821–4830.

4. Schäfer A, Tauch A, Jäger W, Kalinowski J, Thierbach G, Pühler A. Small mobilizable multi-purpose cloning vectors derived from the *Escherichia coli* plasmids pK18 and pK19: selection of defined deletions in the chromosome of *Corynebacterium glutamicum*. Gene 1994, **145:**69-73.
